# MITF in Normal Melanocytes, Cutaneous and Uveal Melanoma: A Delicate Balance

**DOI:** 10.3390/ijms23116001

**Published:** 2022-05-26

**Authors:** Maria Chiara Gelmi, Laurien E. Houtzagers, Thomas Strub, Imène Krossa, Martine J. Jager

**Affiliations:** 1 Department of Ophthalmology, Leiden University Medical Center, P.O. Box 9600, 2300 RC Leiden, The Netherlands; m.c.gelmi@lumc.nl (M.C.G.); lhoutzagers@gmail.com (L.E.H.); 2 Université Côte d’Azur, 06103 Nice, France; thomas.strub@unice.fr (T.S.); imene.krossa@univ-cotedazur.fr (I.K.); 3 Inserm, Biology and Pathologies of Melanocytes, Team1, Equipe Labellisée Ligue 2020, Centre Méditerranéen de Médecine Moléculaire, 06204 Nice, France

**Keywords:** eye, oncology, melanoma, MITF, melanocyte, cutaneous melanoma, uveal melanoma

## Abstract

Microphthalmia-associated transcription factor (MITF) is an important regulator of melanogenesis and melanocyte development. Although it has been studied extensively in cutaneous melanoma, the role of MITF in uveal melanoma (UM) has not been explored in much detail. We review the literature about the role of MITF in normal melanocytes, in cutaneous melanoma, and in UM. In normal melanocytes, MITF regulates melanocyte development, melanin synthesis, and melanocyte survival. The expression profile and the behaviour of MITF-expressing cells suggest that MITF promotes local proliferation and inhibits invasion, inflammation, and epithelial-to-mesenchymal (EMT) transition. Loss of MITF expression leads to increased invasion and inflammation and is more prevalent in malignant cells. Cutaneous melanoma cells switch between MITF-high and MITF-low states in different phases of tumour development. In UM, MITF loss is associated with loss of BAP1 protein expression, which is a marker of poor prognosis. These data indicate a dual role for MITF in benign and malignant melanocytic cells.

## 1. Introduction

Melanocytes, which originate from the neural crest, are located in many areas of the human body, such as the skin and eye. Their most obvious physiological function is to produce melanin, which gives the skin, hair, and eye their characteristic colours. However, melanocytes have been demonstrated to interact closely with other cell types in the skin and to be involved in immune responses, to act as stress sensors and to have neuroendocrine properties [1,2,3]. 

The environment around melanocytes in the skin and in the eye is distinct: skin melanocytes are surrounded by keratinocytes, whereas the neighbours of ocular melanocytes can be fibroblasts, endothelial cells or epithelial cells, depending on the tissue (choroid, iris, ciliary body, or conjunctiva). In the eye, there is another type of pigmented cell, called a retinal pigmented epithelial cell, which is derived from the optic neuroepithelium. Their neighbours are photoreceptor cells.

Transformation of melanocytes leads to melanoma. Cutaneous malignant melanoma represents only 5% of skin cancers but is responsible for 75% of deaths by a cutaneous cancer. Its incidence is 30 to 380 cases per million per year and depends on the geographical location and genetic background [4]. Cutaneous malignant melanoma is epidemiologically linked to exposure to ultraviolet (UV) radiation of the solar light [5,6]. However, the relationship between UVR and cutaneous melanoma is not yet fully understood, given the absence of a UV DNA damage signature in genes relevant to cutaneous melanoma such as *BRAF, NRAS*, or *CDKN2A* (cyclin dependent kinase inhibitor 2A) [7]. UM (uveal melanoma) is the most common primary intraocular malignancy in adults. Although UM is relatively rare compared to cutaneous melanomas, with an incidence of 1 to 9 cases per million per year [8], depending on the geographical location, it contributes to a large proportion of melanoma death rates. Approximately 95% of ocular melanomas occur in the uvea (of which 90% are in the choroid, 6% in the ciliary body, and 4% in the iris), and 5% in the conjunctiva [8,9,10,11]. Driver mutations in choroidal melanomas are mutations in the heterotrimeric G-protein alpha subunits *GNAQ* and *GNA11*, while conjunctival melanomas show more resemblance to skin melanoma with oncogenic mutations in *BRAF* and *NRAS*. 

Fair skin, blond/red hair, light iris colour and nevi are risk factors for both types of melanomas [12]. Risk factors for cutaneous melanoma also include freckles, intense sun exposure and strong skin reactions to sun as well as genetic mutations in a panel of genes such as *CDKN2A*, *MC1R* (melanocortin 1 receptor), *MITF* (microphthalmia-associated transcription factor) and *BAP1* (BRCA1 associated protein 1) [13]. Risk factors for UM include germline mutations in BAP1 [14,15] and single nucleotide polymorphisms (SNP) in pigmentation genes such as *HERC2*/*OCA2* and *IRF4* (interferon regulatory factor 4) [16].

As a light iris colour (blue/grey/green) is especially seen in populations in North-Western Europe and worldwide in people of Northern European ancestry, areas inhabited by these populations have the highest number of UM cases [17]. 

*MITF* plays a critical role in melanocyte development as well as their function, survival, and proliferation and is considered the master gene of melanocyte homeostasis and a critical factor in melanoma biology (as reviewed by Cheli et al. [18] and by Goding and Arnheiter [19]). MITF is a basic helix-loop-helix-zipper (bHLHZip) transcription factor encoded on chromosome 3p12.3-14.1. It encodes multiple isoforms that show ubiquitous or tissue-specific expression. MITF-M is the main isoform of the melanocyte lineage, while MITF-D and MITF-H are detected in adult retinal pigment epithelium (RPE). MITF-H is also expressed in the heart tissue whereas MITF-A is ubiquitously expressed [19]. MITF has been extensively studied in skin melanocytes and cutaneous melanoma over the past decades. 

We reviewed the literature regarding the role and regulation of MITF expression and its function in normal cutaneous melanocytes and compared its expression and function between cutaneous melanoma and UM cells. 

## 2. Normal Regulation

### 2.1. Role of MITF in Skin Melanocyte Development

During embryonic development, pluripotent neural crest cells give rise to non-pigmented melanoblasts. The melanoblasts then migrate from both the dorsolateral and the ventral pathways to the basal layer of the epidermis and to the hair follicles. In the epidermis, melanoblasts differentiate into mature melanocytes and start producing melanin, which is transferred to surrounding keratinocytes to ensure skin pigmentation. Melanocytes are not present at the dermal–epidermal junction of mouse skin except on the ear, tail, nose, and foot pad. The hair follicle contains several different types of melanocytes with different properties in different locations [20,21]. Melanogenically-active melanocytes can be found in three locations (close to the dermal papilla, in the infundibulum and in the sebaceous glands), while melanogenically-inactive melanocytes are located in the stem cell compartment of the hair bulge, in the outer root sheath, and in the peripheral–proximal hair bulb.

MITF plays a key role in melanocyte development. In humans, mutations in *MITF* have been identified in patients with Waardenburg syndrome (WS) type 2A and Tietz syndrome, leading to pigmentation defects such as patches of light skin and iris heterochromia and deafness [22,23]. More recently, mutations in *MITF* have been identified in patients with Coloboma who also show osteopetrosis, microphthalmia, macrocephaly, albinism, and deafness [24]. In mice, heterozygous *MITF* mutants usually do not show a visible phenotype but some alleles can lead to alterations in pigmentation of the skin including white spots on the belly, head, tail, and a reduction in iris pigmentation. In contrast, absence of MITF leads to a complete lack of neural crest-derived pigment cells with not only a lack of pigmentation but also with ear and eye developmental abnormalities [25]. Defects in mast cells and osteoclasts have been observed in homozygotes carrying certain mutant alleles [25,26]. 

The essential role of MITF-M in melanocyte development is demonstrated in the black-eyed white *Mitf*^mi-bw^ mice [27], which harbour the insertion of an L1 retrotransposable element in intron 3, leading to complete repression of MITF-M mRNA expression. These mice exhibit a completely white coat colour due to deficiency of melanocytes in their skin and hair bulbs, with deafness, and black eyes: they lack choroidal melanocytes but their RPE cells are normally developed and pigmented [27,28]. 

MITF-M is also required for melanoblast survival [29,30]. This can be mediated through induction of the anti-apoptotic target genes *BCL-2* (B-cell-lymphoma 2) [31], and of *DICER*, which was shown to be crucial to melanocyte survival through its function in the processing of miRNAs [32].

During embryonic development, melanocytes undergo epithelial-to-mesenchymal transition (EMT) in order to delaminate from the neural tube [33]. EMT consists of a shift from an epithelial-like, less-motile phenotype to a mesenchymal-like, more-motile phenotype. EMT is regulated by several transcription factors (EMT-TFs) among which are SNAI1 (snail family transcriptional repressor 1), SNAI2 (snail family transcriptional repressor 2), zinc-finger E box-binding homeobox 1 and 2 (ZEB1 and ZEB2), and twist-related protein 1 (TWIST1) [34]. Since melanocytes are not epithelial in nature, but originate from the neural crest, we may refer to EMT in this cell type as pseudo-EMT. A further interesting function of MITF is its interaction with pseudo-EMT, which is involved in pathological processes such as cancer. A mutation in *SNAI2* has been observed in two patients with Waardenburg syndrome type 2, and MITF has been shown to induce the transcription of *SNAI2*, the promoter of which has a potential MITF binding site [35]. 

### 2.2. Role of MITF in Melanocyte Function (Melanocytes and Melanin)

MITF has an important role in melanocyte function, since it is involved in several processes, such as melanin synthesis and pigmentation, and melanocyte structure, survival, proliferation, and differentiation.

Skin colour is related to the type of melanin, the size, and the number of melanosomes, and how the melanocytes are dispersed in the keratinocytes. Melanogenesis is under complex regulatory control by multiple agents, interacting via pathways activated by receptor-dependent and -independent mechanisms, in hormonal, auto-, para-, or intracrine fashion or by UV radiation [36]. The most pro-pigmentation pathway involves the binding of α-MSH (α-melanocyte stimulating hormone), produced by the keratinocytes, on its MC1R, which is expressed by melanocytes. Mechanistically, α-MSH binding to MC1R triggers elevation of cAMP and activation of the PKA/CREB (cAMP response element)-signalling pathway that stimulates transcription of a variety of downstream targets, including MITF [37,38].

Melanin is synthetized in specialized lysosomal-related organelles called melanosomes. They can produce two types of melanin: eumelanin is a brownish-black insoluble polymer associated with protection against UV-mediated DNA damage, while pheomelanin is a yellowish-red soluble polymer associated with the induction of genotoxic stress [39]. The main enzymes that drive melanogenesis are TYR (tyrosinase), TYRP-1 (tyrosine-related protein-1) and DCT (dopachrome tautomerase, also known as tyrosine-related protein-2, TYRP-2). The expression of these melanogenic enzymes is regulated by MITF through a direct interaction with an E-box/M-box in their promoters [18,40,41,42]. The activation of the *DCT* promoter by MITF happens in cooperation with CREB, SOX10 (SRY-box transcription factor 10), or LEF-1 (lymphoid enhancer binding factor 1) [43,44,45]. PAX3 (paired box 3) has been shown to have a dual role: on the one hand it antagonises the MITF-mediated stimulation of DCT, while on the other hand, it enhances MITF expression [46]. Specific *MC1R* variants have been associated with red hair and a high pheomelanin content [47], but not with iris pigmentation [48]. The cAMP-dependent signalling pathway also operates through the expression of PKCβ (protein kinase C beta) via MITF, which has been reported to phosphorylate and activate TYR [49]. 

**Figure 1:**
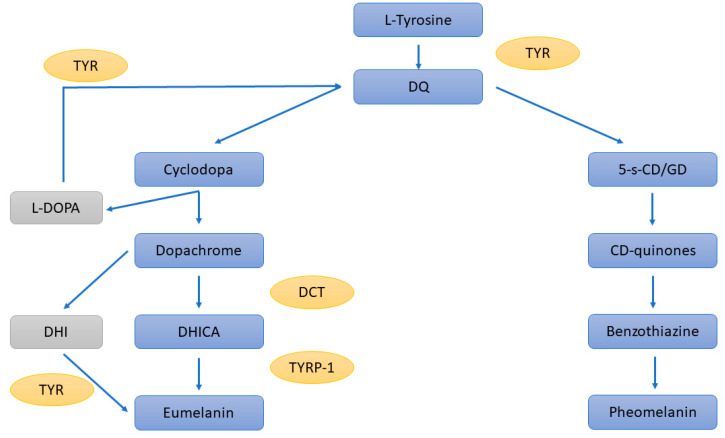
Schematic representation of the cascade of melanin synthesis. TYR = tyrosinase, DCT = dopachrome tautomerase, TYRP-1 = tyrosinase-related protein 1, L-DOPA = dihydroxyphenylalanine, DHI = dihydroxyindole, DHICA = dihydroxindole-2-carboxylic acid, 5-s-CD/GD = 5-S-cysteinyldopa or glutathionyldopa, CD-quinones = cysteinyldopa-quinones.

The production of melanin starts with the oxidation of L-tyrosine to L-DOPA and then to DOPAquinone (DQ) by TYR; this is a rate-limiting step (Figure 1). DQ can follow two distinct pathways, leading to either the production of eumelanin or pheomelanin: it can undergo cyclisation and give rise to cyclodopa, followed by a redox reaction that produces dopachrome and dihydroxyphenylalanine (DOPA). While DOPA is converted back to DQ by TYR, dopachrome either undergoes decarboxylation to dihydroxyindole (DHI) or is converted into dihydroxindole-2-carboxylic acid (DHICA) by DCT. Both DHI and DHICA are subsequently oxidised to eumelanin. In the presence of cysteine or glutathione, DQ is converted to 5-S-cysteinyldopa or glutathionyldopa, which are then oxidised to benzothiazine and finally to pheomelanin [50].

In the skin, each melanocyte is in contact with on average 36 keratinocytes, thereby forming the epidermal melanin unit. Mature melanin-containing melanosomes are thought to be transported to the melanocyte’s dendrite tips in an actin- and microtubule-dependent process that is regulated by Rab27a, melanophilin, and myosin Va [51,52,53,54] and subsequently to be transferred to keratinocytes to ensure homogeneous skin pigmentation. These processes also implicate MITF, which regulates the transcription and expression of genes involved in melanosome functioning (*GPR143/OA1*, *SILV/PMEL/gp100*, *MLANA/MART-1* [18,55,56]), and transport (Rab27). The mechanisms governing melanosome transfer and positioning are still debated and poorly understood [57,58].

It is worth noting that L-tyrosine and L-dihydroxyphenylalanine (L-DOPA), in addition to serving as substrates and intermediates of melanogenesis, are thought to regulate other melanocyte functions through action on specific receptors or through non-receptor-mediated mechanisms [36,59].

At the same time, there is increasing evidence that melanocytes may have other functions in the skin. Indeed, being capable of secreting a wide range of signalling molecules (cytokines, POMC peptides including α-MSH, catecholamines, and NO), melanocytes could impact on a range of skin cells to maintain epidermal homeostasis [2,60]. 

As mentioned previously, pigmentation and melanin production are not the only functions of MITF in melanocytes. MITF is also involved in melanocyte survival, proliferation, and cell cycle. It is a positive regulator of the anti-apoptotic gene *BCL2* (BCL2 apoptosis regulator) both in normal melanocytes and in human cutaneous melanoma cells [31], and of *BIRC7* (baculoviral IAP repeat containing 7) in melanoma cells [61]. Levy et al. reported that MITF regulates DICER, which is a regulator of miRNA processing and is necessary for melanocyte development and survival [32]. Moreover, Du et al. reported that MITF controls the expression of *CDK2* (cyclin dependent kinase 2) both in normal melanocytes and in melanoma cells, and that this interaction is relevant for the growth of melanoma cells [62]. Among the targets of MITF we find factors involved in cell cycle-regulation as well: MITF can increase expression of p21 (CDKN1A) and p16 INK4a (CDKN2A), thus causing cell cycle exit and impairing cell growth in melanocytes and in fibroblasts treated with MITF [63,64]. Moreover, both p21 and p16INK4a induce fibroblasts to acquire features typical of melanocyte differentiation. 

**Figure 2:**
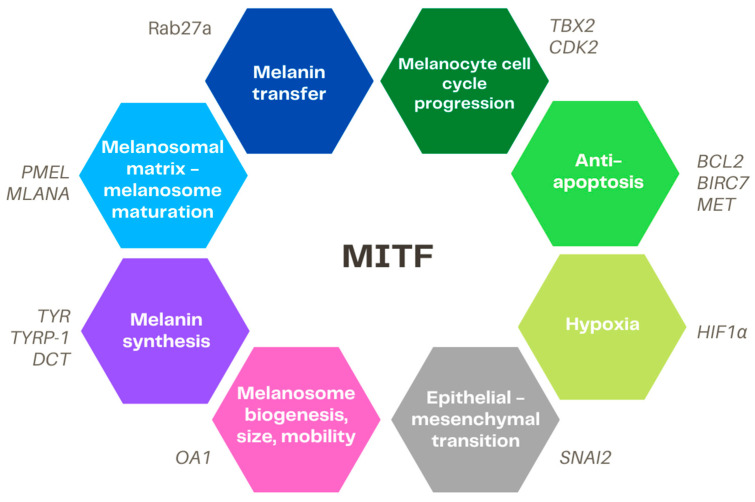
Overview of the main targets of MITF. MITF = microphthalmia-associated transcription factor, PMEL = premelanosome protein, MLANA = Melan-A, Rab27a = RAB27A, member RAS oncogene family, TBX2 = T-box transcription factor 2, CDK2 = cyclin dependent kinase 2, BCL2 = BCL2 apoptosis regulator, BIRC7 = baculoviral IAP repeat containing 7, MET = MET proto-oncogene, receptor tyrosine kinase, HIF1α = hypoxia-inducible factor 1 alpha, SNAI2 = snail family transcriptional repressor 2, OA1 = ocular albinism 1, TYR = tyrosinase, DCT = dopachrome tautomerase, TYRP-1 = tyrosinase-related protein 1.

A summary of the main MITF targets is presented in Figure 2.

### 2.3. Regulation of MITF Expression in Normal Skin Melanocytes

**Figure 3:**
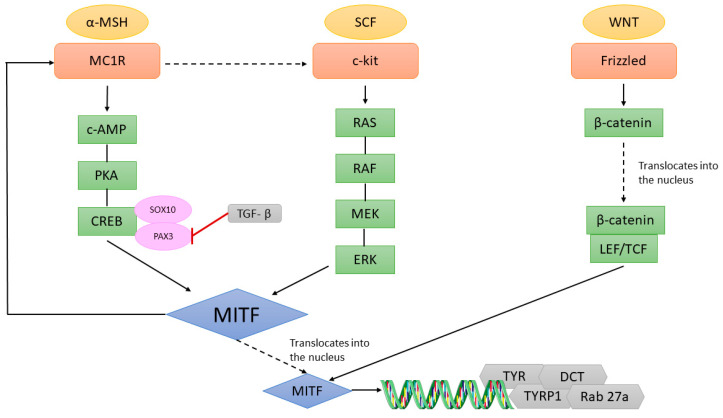
Schematic representation of the pathways that lead to MITF activation. α-MSH = α-melanocyte-stimulating hormone, MC1R = melanocortin 1 receptor, c-AMP = cyclin adenosine monophosphate, PKA = protein kinase A, CREB = cAMP response element, SOX10 = SRY-Box transcription factor 10, PAX3 = paired box 3, SCF = stem cell factor, ERK = extracellular signal-regulated kinase, LEF/TCF = lymphoid enhancer-binding factor/T cell factor, MITF = microphthalmia-associated transcription factor, TYR = tyrosinase, DCT = dopachrome tautomerase, TYRP-1 = tyrosinase-related protein 1.

The *MITF-M* promoter is upregulated by the transcription factors PAX3 [65] and SOX10. Mutations in *PAX3* are responsible for WS types 1 and 3 and mutations in *SOX10* for WS type 4 [66]. Moreover, Wnt signalling is required for the expansion and for determining the fate of neural crest derivatives, including melanocytes during early development [67,68,69]. Wnt-3a expression is detected at embryonic day 7.5 [70], which precedes that of MITF expression in neural crest cells (9.5–10.5 days) [30]. Wnt3 regulates β-catenin, which translocates into the nucleus, where it then interacts with LEF/TCF (lymphoid enhancer-binding factor/T cell factor) and stimulates MITF transcription [70,71]. A study on mouse hair follicles and skin showed that *ZEB2* knockout greatly impaired melanocyte differentiation and decreased the expression of *MITF* and other pigment-related genes in mouse melanocyte, suggesting that *MITF* might be a direct target of EMT factors [72]. As already mentioned, MC1R, through activation of the PKA/CREB module, is an important regulator of MITF transcription in the skin. SOX10 enhances MITF expression and it cooperates with CREB and PAX3, which is particularly active in early melanocyte development and is inhibited by TGFβ (transforming growth factor β) [66,73,74,75]. These transcription factors are thus regulators of MITF, while they themselves are regulated by MITF as well. Knockout of the receptor tyrosine kinase *KIT* in melanocytes causes a severe white spotting phenotype [76]. Mechanistically, SCF (stem cell factor) binds to c-Kit and stimulates the MAPK (mitogen activated protein kinase) and PI3K (phosphatidyl inositol-3-kinase) pathways. Activation of ERK (extracellular signal regulated kinase) and subsequent phosphorylation of MITF on Ser73 increases MITF activity [77,78,79] (Figure 3). Ser73 phosphorylation is a priming site for GSK3 (glycogen synthase kinase 3) phosphorylation on Ser69 which prevents MITF nuclear export, while phosphorylation on Ser409 may prime for phosphorylation of S405, S401, and S397 by GSK3 [80,81]. Thus, BRAF/MAPK and PI3K/GSK3 signalling converges to control MITF nuclear export and activity. Finally, miR-218 suppressed melanogenesis in human pigmented skin, through MITF repression [82]. In conclusion, in cutaneous melanocytes, MITF expression is regulated through several signalling pathways, both at the mRNA and protein level, in which epigenetic regulation also plays a role.

## 3. MITF in Cutaneous Melanoma

Cutaneous malignant melanomas are heterogeneous in nature, comprising several cell subpopulations with distinct transcriptomic signatures and behaviours [83]. Melanomas carrying different genetic alterations have different clinical features and different relation with sun exposure. A study of the TCGA database divided melanomas into four genomic subtypes: BRAF mutated (more common in younger patients, associated with intermittent sun exposure), NRAS mutated (more common in older patients, associated with chronic UV damage), NFI mutated (present in acral and mucosal melanomas, associated with chronic UV damage), and triple wild type (lacking a UV signature, including uveal melanoma) [84]. Other mutations and genetic alterations are known to be present in cutaneous melanoma, such as CDKN2A mutation, PTEN loss, and amplification of MITF, EGFR, CCDN1, cMET, and cKIT [85,86,87]. Epigenetic modifications such as changes in the DNA methylation status, chromatin remodelling and non-coding RNA regulation may also influence the behaviour of melanoma cells and the response to therapy [88]. Two main cell populations have been identified: a fast-replicating population with low invasive potential (with high MITF expression) and a slowly proliferating population with high invasive potential (with low MITF expression). MITF-low cutaneous melanoma cells display a higher expression of stem cell markers (OCT4 and NANOG) and are able to produce larger tumours when injected into nude mice [89]. However, both MITF-low and MITF-high cells can give rise to tumours, which then contain both types of cells [89,90]. This finding served as evidence that these two populations of cells are not genetically determined but that the proliferative or invasive potential is likely to be the result of a phenotype switch [89,91]. While it is not yet fully clear what determines the switch between the two phenotypes, factors such as hypoxia and nutrient starvation have been implicated and MITF is thought to play a key role [89,92]. 

### 3.1. Cell Proliferation

MITF has been shown to enhance or to inhibit proliferation. On one hand, MITF may behave as a melanocyte specific oncogene. MITF is expressed in about 80% of human melanomas (since it is not frequently expressed in desmoplastic melanomas) [93,94]; it is amplified in 10% of primary and 20% of metastatic cutaneous melanomas and its expression correlates with decreased 5-year overall patient survival [95]. Furthermore, a rare germline variant in the *MITF* gene (E318K variant) has been linked to a high total nevus count and an increased risk of cutaneous melanoma [96,97]. Another illustration of MITF’s role in proliferation is its ability to control the expression of the cell cycle regulators. MITF is known to regulate transcription and expression of the cyclin dependent kinases *CDK2* (cyclin dependent kinase 2) and *CDK4* (cyclin dependent kinase 4) [95,98], of *TBX2* (T-box transcription factor 2, a transcription factor of the T-box family), that in turn blocks senescence through repression of p21 and p19 [99,100,101,102], and of several genes involved in mitosis [103]. Moreover, MITF exerts a positive control over cell cycle progression through degradation of the growth inhibitor p27 on the one hand, while at the same time it inhibits invasiveness. Both of these functions are carried out through the *DIAPH1* (diaphanous related formin 1) gene [104]. MITF also drives expression of a large subset of genes involved in lysosome biogenesis and functioning [81], which triggers increased activity of the lysosome-bound mTORC1 (mTOR complex 1) and global protein synthesis [105]. Likewise, MITF controls expression of the metabolic factor *PGC1a* (PPARG coactivator 1 alpha) [106,107]. Enhanced levels of protein synthesis and metabolic activities could allow cancer cells to cope with the metabolic demand related to the high proliferative rate associated with MITF. Consequently, MITF knockdown in human cutaneous melanoma cell lines promotes a growth arrest through induction of a senescence-like phenotype [108]. On the other hand, MITF has been reported to exert an antiproliferative effect in cutaneous melanoma cells, essentially via p21 regulation [63]. 

### 3.2. Cell Survival

MITF has been shown to promote cell survival in melanoma cells through several mechanisms. MITF binds E-boxes on the promoters of anti-apoptotic target genes *BCL2, BCL2A1* (BCL2 related protein A1) [109] and *BIRC7* (baculoviral IAP repeat containing 7) [31,61] and participates in transactivation of the receptor tyrosine kinase *MET*, thereby increasing the anti-apoptotic effect of the MET ligand HGF (hepatocyte growth factor) [110]. Under oxidative stress conditions, MITF activates APE1/Ref1 (apurinic-apyrimidinic endonuclease1⁄redox factor-1), which is a protein involved in DNA repair and in redox regulation [111]. HIF1α is one of the targets of APE1/Ref1 [112]. HIF1α is also a direct target of MITF. Aligned with this, enhanced *HIF1α* expression impaired staurosporin-induced cell death in cutaneous melanoma cells [113].

### 3.3. Epithelial-Mesenchymal Transition and Motile Ability

**Figure 4:**
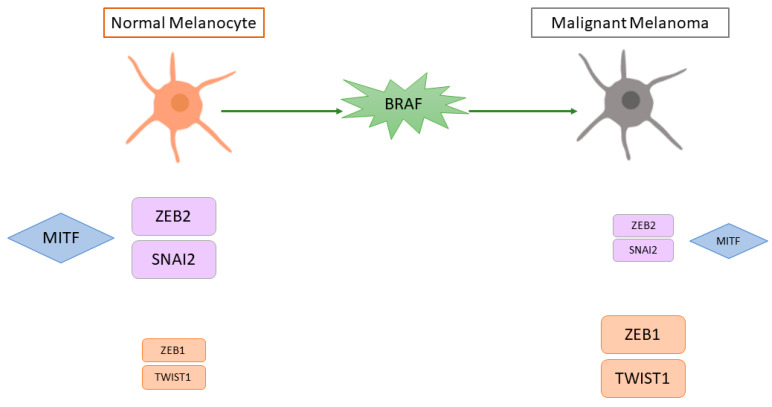
Schematic representation of the putative effect of BRAF mutation on pseudo-epithelial-mesenchymal transition and MITF in cutaneous melanoma. MITF = microphthalmia-associated transcription factor, SNAI2 = snail family transcriptional repressor 2, ZEB2 = zinc finger E-box binding homeobox 2, ZEB1 = zinc finger E-box binding homeobox 1, TWIST1 = twist family BHLH transcription factor 1, BRAF = B-Raf proto-oncogene, serine/threonine kinase. The size of the boxes represents the level of expression (higher expression = bigger box; lower expression = smaller box).

EMT is a complex process in which epithelial cells acquire the characteristics of invasive mesenchymal cells. Melanoma tumour progression and metastasis formation involves a pseudo-EMT process (given the non-epithelial nature of melanoma cells) in which MITF is also involved. Normal cutaneous melanocytes have a high expression of SNAI2 and ZEB2 and a low expression of ZEB1 and TWIST1, while malignant cutaneous melanomas have low SNAI2 and ZEB2 and high ZEB1 and TWIST1 [114]. Survival analysis in cutaneous melanoma patients showed that high TWIST1 and ZEB1 expression was associated with a shorter metastasis-free survival. Moreover, in vitro BRAF activation caused a switch from a ZEB2high/SNAI2high/ZEB1low/TWIST1low state (similar to normal melanocytes) to a ZEB2low/SNAI2low/ZEB1high/TWIST1high state and MEK inhibitors reversed this switch [114]. Gene expression profiling revealed that cell lines with high *ZEB1* and *TWIST1* had a de-differentiated gene signature characterised by an upregulation of invasion-associated and TGFβ-regulated genes and downregulation of *MITF* and its target genes [114]. The role of ZEB2 was confirmed in another study, in which human primary cutaneous melanoma samples with high nuclear ZEB2 staining were associated with a better prognosis than tumours with low nuclear ZEB2 staining [72]. *ZEB2* knockdown in mouse melanoma cell lines led to a decrease in *MITF* and its target genes and an increase in *ZEB1*, thereby leading to a more invasive phenotype. A subsequent study by the same group confirmed that ZEB2 is associated with a proliferative gene signature that includes MITF, while ZEB1 is associated with an invasive gene signature [115]. A schematic representation of the role of MITF in pseudo-EMT is presented in Figure 4.

In conclusion, MITF’s role in melanoma cells is important and complex. The rheostat model proposed by the group of Goding provides explanation to the apparent paradox that MITF controls or represses the proliferation or the motile ability of melanoma cells [104]. MITF expression levels are important but by far not the only criterion in physiology and pathology of melanocytes and melanoma cells. MITF activity also depends on its post-translational modifications (phosphorylation, SUMOylation, ubiquitination) and co-factors (such as p300, BRG1, β-catenin). Hence, cells with low MITF levels are poorly proliferative (likely due to increased p27 expression), dedifferentiated, more mesenchymal and motile [104], whereas cells with high MITF activity are differentiated and growth-arrested in part through p21 induction [63]. Supporting this model, positive MITF staining in the primary tumour was associated with a better survival and lower rates of lymph node involvement in cutaneous melanoma patients [116,117].

### 3.4. Regulation of MITF in Cutaneous Melanoma

Multiple factors and stimuli have been shown to control MITF-M expression in cutaneous melanoma cells. As previously described for skin melanocytes, CREB, PAX3, SOX10, and the Wnt/β-catenin module are also well known upstream regulators of MITF in cutaneous melanoma cells [19]. In contrast, ATF4 (activating transcription factor 4), and JUN, which both integrate stress signals, repress *MITF* expression and trigger cutaneous melanoma cell dedifferentiation [118,119,120]. Another gene that is able to repress MITF is *BRN2,* which mediates melanoma cell invasion [121]. This gene is negatively regulated at the post-transcriptional level by miR-211, which is in turn upregulated by MITF. miR-211 is not the only miRNA involved in the regulation of the invasiveness of cutaneous melanoma cells [122]. Data regarding miR-182 have shown conflicting results, with some authors reporting it to be upregulated in advanced melanoma and other authors stating that it is downregulated in cutaneous melanoma samples [123]. Changes in the tumour microenvironment, such as hypoxia or nutrient starvation, may also cause a decrease in MITF expression and greater invasiveness [92,118,124]. Cutaneous melanoma cells exposed to hypoxia have a higher HIF1α expression and lower MITF expression and give rise to larger tumours and more frequent metastases [92]. The downregulation of MITF caused by hypoxia is dependent on HIF1α, which through the transcription factor Bhlhb2 represses the MITF promoter [92,125]. Melanogenesis has been shown to generate an immunosuppressive and mutagenic environment and alters the glycolytic metabolism through HIF1α induction [126,127]. Likewise, glutamine starvation reduces MITF level through ATF4 induction [118]. Additional cues such as TNFα (tumour necrosis factor α) and TGFβ are likely factors that induce the phenotype switch in cutaneous melanoma cells. TNFα can also activate ATF4 and JUN, resulting in dedifferentiated cutaneous melanoma cells [118,119,120]. TGFβ antagonizes MITF function, represses pigmentation and stimulates the motile ability of cutaneous melanoma cells [128,129]. 

### 3.5. Clinical Relevance

Both immune checkpoint inhibitors (ICI) and molecularly targeted therapy with BRAF and MEK inhibitors (BRAF/MEKi) are standard options for patients with *BRAFV600*-mutated, unresectable, or metastatic melanoma. Options in BRAF wild-type melanoma are limited to ICIs. Despite the progress brought by these treatments, about 50% of the patients reach a therapeutic dead end due to primary or secondary resistance. As mentioned above, cutaneous melanomas are heterogeneous tumours comprised of cells with distinct transcriptomic signatures driving specific behaviours. The two most studied types of melanoma cells are those with a proliferative or invasive phenotype. Due to their high intrinsic plasticity, cutaneous melanoma cells can switch back and forth between these two phenotypes. This plasticity is thought to create intratumour heterogeneity which plays a key role in treatment failure and relapse. Thus, it is of paramount importance to better understand the features of these cell subpopulations to improve treatment efficiency. MITF is thought to be the very determinant of melanoma cell plasticity [89,130]. Genomic amplification of the MITF target, *BCL2A1*, has been implicated in resistance to BRAF inhibitors by Haq et al. [109]. Moreover, macrophage-derived TNFα, through increased MITF expression, provides resistance to MAPK pathway inhibitors [131]. Consequently, inhibition of MITF, by introduction of a dominant-negative MITF mutant in melanoma cells with MITF amplification, or by blocking the TNFα signalling with IκB kinase inhibitors, increased susceptibility of cutaneous melanoma cells to chemotherapeutic agents or MAPK pathway inhibitors, respectively [95,131]. These observations suggest that MITF inhibition may represent an option to increase therapy efficacy. However, low MITF expression in cutaneous melanoma cells has also been linked to phenotype switching and drug resistance. Indeed, MITF knockdown in melanoma cells leads to a senescent-like phenotype that is associated with NFkB (nuclear factor kappa-light-chain-enhancer of activated B cell) activation and production of an inflammatory secretome (CCL2, IL6, IL1). This secretome favours a more mesenchymal phenotype, thereby favouring melanoma progression and metastatic dissemination [132,133]. Aligned with these findings, Konieczkowski et al. showed that BRAF-mutated melanoma cells with intrinsic resistance to MAPK inhibitors display a low MITF and high NFkB expression [134]. Likewise, NFkB induction by TNFα led to a decrease in MITF expression and conferred resistance to MAPK inhibitors [134]. MITF low cells are also associated with an increase in the expression of stem cell genes and reprogramming to a more invasive cell state. Interestingly, MITF activity has been recently shown to be regulated by a direct interaction with RAF proteins in melanoma cells [135]. By triggering a partial relocation of MITF in the cytoplasm, this interaction might reduce nuclear concentrations of MITF, thereby impacting phenotype switching and therapy efficacy. Melanisation level can also affect the therapy and the clinical outcome of advanced pigmented melanomas [136,137,138].

As highlighted in a review by Ballotti et al., in addition to resistance to targeted therapy, downregulation of *MITF* may be also responsible for resistance to immunotherapy [139] and subsequent melanoma progression [139]. Inflammatory signals produced by low MITF melanoma cells or by the microenvironment can induce de-differentiation and the consequent loss of melanocyte-specific surface antigens [139,140]. Collectively, these observations are in agreement with Müller et al. reporting the existence of two types of resistant cell lines: one with high or normal levels of MITF and one with low MITF level [141]. Nevertheless, they showed that the cell lines with low MITF levels are more resistant to a wider panel and higher concentrations of MAPK pathway inhibitors than the ones with high MITF [141]. 

## 4. MITF and the Eye

In the eye, pigment producing cells can be found in different locations. Melanocytes in the choroid, iris, ciliary body, and conjunctiva are derived from the dorsal neural crest and follow the same path as epidermal melanocytes for development, migrating along the dorsolateral pathway from the neural crest to the uvea or conjunctiva. Studies in mice showed that melanoblasts in the eye reach the choroid at an early embryological stage and increase in density in the early postnatal phase, acquiring a perivascular distribution. The RPE, which is composed of a single layer of cells containing pigment granules, is derived from the optic vesicle [142].

The regulation of eye colour is far less understood than skin colour. Both eye melanocytes and RPE cells contain melanin-filled melanosomes. More important than the melanin ratio is the density of melanosomes in the stromal melanocytes, with density increasing from blue to brown eyes [143,144]. A study by Wakamatsu et al. showed that iridal and choroidal melanocytes from eyes with a dark iris had significantly higher total melanin content and a higher eumelanin/pheomelanin ratio than melanocytes from eyes with a light iris [145]. Iris tissue from light eyes also showed a slightly higher pheomelanin content compared to dark eyes, but it did not reach statistical significance. There is no compelling evidence suggesting that the pigment-containing structures are secreted and/or transferred to adjacent cells or that RPE cells produce pheomelanin.

Genetic defects in expression of some melanogenic enzymes (TYR, TYRP1) or other melanin synthesis regulators (P protein, SLC45A2,) result in oculocutaneous albinism (OCA1-OCA4) and visual function impairment, demonstrating the importance of melanin for proper ocular function [146,147]. Single-nucleotide polymorphisms (SNPs) in other genes including *HERC2/OCA2, LYST* (lysosomal trafficking regulator)*, IRF4,* and *SLC24A4* (solute carrier family 24 member 4) have also been implicated in determining eye colour [148,149,150,151]. Simcoe et al. published the results of a very large genome-wide association study involving two European cohorts and two smaller Asian cohorts and reported 50 novel genetic loci related to eye colour (alongside the previously-known loci). Interestingly, *MITF* and *DCT* were among the genes containing significantly-associated SNPs [152]. 

### 4.1. Expression of MITF in Ocular Tissues

Several transcription factors are involved in the specification of intraocular tissues and MITF plays a crucial role. Initially, MITF-M has been shown to be expressed in melanocytes but not in RPE cell lines [153] while studies have shown important roles of *MITF-H* and *MITF-D* in mouse RPE specification and differentiation [39,154]. MITF-H and MITF-D are controlled by the retina-specific transcription factor CHX10 [154]. Absence of H- and D-MITF in *MITF* red-eyed white *(MITF*^mi-rw^) mutant mice, which may present with low and variable levels of pigmentation in the peripheral iris, exhibit severe RPE developmental abnormalities [154]. It is worth noting that MITF-H has the ability to compensate for reduced RPE pigmentation induced by MITF-D loss [155]. Other *MITF* mutations can cause RPE and retinal dysfunction, degeneration and thus worsen vision [156]. Isoforms MITF-A and MITF-J are more ubiquitous, as they are expressed in melanocytes and RPE cells as well as in retinal cells, but they do not seem to have a significant impact on eye development [154,157] (Table 1). More recently, Esumi and coworkers uncovered that differentiated human adult RPE cells expressed *MITF-M* [158]. Nevertheless, the black-eyed white *Mitf*^mi-bw^ mutant mice lack *MITF-M* expression and melanocytes in the skin and choroid, but retain RPE pigmentation [27,159], indicating that *MITF-M* is not essential for RPE development or differentiation. Therefore, MITF-M might be functional in human adult RPE, although its functions in RPE cells and whether they are distinct from those of the other MITF isoforms remain to be determined.

It is worth noting that mice with the *MITF*^mi-bw^ mutation developed a thinner choroid, containing only one vascular layer, as opposed to the normal two layers, indicating that MITF has been observed to have a role in the development of choroidal vessels [160].

### 4.2. Uveal Nevi

In the choroid, melanocytes that acquire mutations in either *GNAQ, GNA11*, or *CYSLTR2* can give rise to nevi [161,162]. Usually, cutaneous nevi do not carry these mutations, being characterised by *BRAF* and *NRAS* mutations [163,164]. However, somatic mutations in *GNAQ*, *GNA11,* or *CYSLTR2* can be found in dermal blue nevi [165,166]. These mutations are not per se able to induce melanocyte transformation and melanoma development. 

The risk of developing a UM is increased in people with an oculodermal pigment aberration, known as nevus of Ota (1 in 400 compared to 1 in 1300 in the general population) [167]. The nevus of Ota may harbour mutations in *GNAQ* but a study on one patient also revealed the presence of a *BAP1* mutation in the primary tumour and *TP53* (tumour protein 53) mutations in a local recurrence [168].

### 4.3. MITF in Ocular Melanoma

It seems eye melanomas are derived exclusively from neural crest melanocytes but never from the neuroepithelium-derived RPE or the posterior layer of the iris. The molecular mechanisms underlying eye melanocyte transformation, especially in the choroid and ciliary body, are not known. In both conjunctival and iris melanoma, molecular findings support the occurrence of UV-induced DNA damage, indicating a link between solar radiation and development of these tumours [169,170]. Clinically and histologically, conjunctival melanomas, which harbour mutations in *BRAF* and *NRAS*, show greater similarity to cutaneous melanoma than does UM. Like choroidal nevi, a UM usually carries either a *GNAQ* or a *GNA11* mutation [171,172]. When a second mutation occurs, it usually involves one of three genes: *BAP1, EIF1AX*, or *SF3B1* [8,173]. 

BAP1 is a high-risk locus in UM and germline *BAP1* mutations increase the risk of developing UM and other tumours such as melanocytic skin tumours, mesothelioma, and renal cell carcinoma (known as the BAP1-predisposition syndrome) [8,174]. Alongside BAP1, some other low-risk loci have been linked to the risk of developing UM: specific SNPs in the pigmentation genes *HERC2* and *OCA2* are associated with a lower risk, and *IRF4* is associated with a higher risk, and deleterious germline mutations of *MBD4*, which give rise to UM with a hypermutator phenotype [8,16].

Only a few studies analysed the presence and function of MITF in UM and, as for cutaneous melanoma, its role seems to be complex: some studies classify MITF as pro-oncogenic, and others mention its expression as a feature of low-risk tumours. Mouriaux et al. showed that 65% of UM were positive for nuclear MITF staining. They found a negative association between MITF staining and tumour pigmentation but a positive correlation between MITF staining and proliferative activity [175]. These observations indicate that, like in cutaneous melanoma, MITF might be linked to the proliferative ability of UM cells. Hippo-YAP/TAZ also emerged as an important signalling pathway downstream of GNAQ/11 that controls UM cell proliferation. Downstream of this module lies PAX3, which controls MITF expression [176]. As such, YAP inhibition suppressed the growth of UM [177,178]. In contrast, MITF has been shown to increase p16 expression in UM, where *CDKN2A* mutations have rarely been described, supporting the idea that MITF can induce cell cycle arrest and behave as a tumour suppressor gene [64]. Similar to the situation in cutaneous melanoma [122,123], miRNAs and epigenetic mechanisms are involved in MITF regulation in UM cells: miR-137 through downregulation of MITF, MET, and CDK6 (cyclin dependent kinase 6) and miR-182 through inhibition of MITF, MET, BCL2, and cyclin D2, causing G1 cell cycle arrest, thereby reducing the number of metabolically-active UM cells [179,180]. In addition, miR-182 (which is activated by p53) causes apoptosis of UM cells [180]. Increase in miR-137 by the DNA hypomethylating agent 5-aza-2′-deoxycytidine or the histone deacetylase inhibitor trichostatin A (TSA) also represents a therapeutic opportunity to impair UM cell proliferation through MITF inhibition [179]. However, as in cutaneous melanoma, MITF inhibition might be associated with a stem cell-like phenotype. Matatall et al. showed that *BAP1* knockdown was associated with an increase in expression of stem cell markers (*NANOG*), and loss of pigmentation markers (*MITF, TYR*, and *DCT*) and with the capacity of UM cells to form anchorage-independent colonies. These data suggest that BAP1 loss induced a dedifferentiated and a stem-like phenotype in UM cells, although this remains to be fully demonstrated [181]. Like cutaneous melanoma, MITF inhibition may activate the NFkB pathway. Stimulation of the NFkB pathway has been reported to favour tumour aggressiveness in UM cells [182]. A comparison of gene expression in normal choroidal melanocytes and primary UM showed downregulation of MITF target genes involved in pigmentation (*TYRP1*, *DCT*) and the detection of stemness markers including *OCT4* in primary UM cells [183]. MITF high and MITF low melanoma cells can coexist in a UM and contribute to intra-tumoural heterogeneity, as has recently been described [184,185,186]. 

### 4.4. MITF as a Therapeutic Target

Currently, there are no available direct MITF inhibitors. Therefore, it is important to continue to understand how MITF is regulated and functions in order to identify potential upstream regulators and/or downstream effectors that may be targeted. In line with that, MITF has been reported as a critical downstream target of the HAT (histone acetyl transferase) p300 promoting human cutaneous melanoma growth [187]. A small molecule p300/CBP inhibitor dramatically reduced the number of human cutaneous melanoma cells in vitro. However, inhibitors of HDAC (histone deacetylase) have also been shown to decrease MITF expression and to inhibit tumour growth in a human cutaneous melanoma xenograft model [188]. Aligned with that, the HDAC trichostatin A (TSA) increases miR-137, which in turn reduces MITF expression in uveal melanoma cells [179]. A recent study showed that both the HDAC inhibitor ACY-1215 and a small molecule inhibitor of the MITF pathway (ML329) were able to reduce the proliferation of a metastatic UM cell line in vitro and caused regression of tumours derived from this cell line in zebrafish, but did not influence tumour dissemination [189]. Moreover, UM cells treated with ACY-1215 showed a concomitant decrease in the protein expression of MITF and several of its targets [189]. This apparent contradiction, i.e., inhibition of HAT and HDAC leading to reduction of MITF activity, remains to be elucidated. The DNA hypomethylating agent 5-aza-2′-deoxycytidine, which is *FDA* approved for some cancer treatments, has also been described to increase miR-137 in uveal melanoma cells and thus could be therapeutically exploited [179].

However, data connecting MITF expression with a highly proliferative, yet more differentiated and less invasive phenotype raise the question of whether downregulating MITF, which may trigger a phenotype switch to a slowly-proliferating and highly-invasive melanoma cell state, will actually be beneficial in the long term.

## 5. Conclusions

MITF has several functions in normal melanocytes: it promotes melanocyte development, differentiation, proliferation, and survival. Its role in the biology of eye melanocytes has been less studied but mouse models show that it plays a key role in visual function. Although not yet fully defined, MITF has roles in melanoma development and progression. Its activity depends on its level of expression, cofactors, and post-translational modifications as proposed by Goding [19]. 

Given that cutaneous and uveal melanoma are governed by mutations in different genes and are found in different environments, the role of MITF might differ between skin and eye melanomas, although we see many similarities. 

## Figures and Tables

**Table 1:** Distribution of MITF isoforms in eye tissues.

Isoform	Location in the Eye
MITF-A	Retina, RPE, choroid
MITF-D	RPE
MITF-H	RPE
MITF-J	Retina, RPE, choroid
MITF-M	Choroid and iris, variable results in RPE
